# Supplementary material for: Enhanced Complement Expression in the Tumor Microenvironment Following Neoadjuvant Therapy: Implications for Immunomodulation and Survival in Pancreatic Ductal Adenocarcinoma
Source: Res Sq. 2024 May 13:rs.3.rs-4104258. Preprint. [Version 1] doi: 10.21203/rs.3.rs-4104258/v1 (PMC11118688; doi:10.21203/rs.3.rs-4104258/v1)
Supplement: 1 — Supplementary Figure 1. The overall experimental Design. Supplementary Figure 2. Differentially Expressed Genes (DEGs) in carcinoma AOIs versus TME AOIs in naïve group and NAT-group. Supplementary Figure 3. Immune cell distribution in carcinoma and TME AOIs across the study cohort. The TME AOIs exhibit significantly greater abundance and diversity of immune cells compared to the carcinoma AOIs. Supplementary Figure 4. Paired analysis of pre-NAT biopsy and post-NAT resection from four patients. A. Among the four patients (14, 15, 16, 17) for whom both the pre-NAT biopsy and post-NAT resection were available, two of them exhibited NAT-induced upregulation of C3 complement level in TME, while the other two patients showed no increase. B. The two patients with NAT-induced upregulation of TME C3 complement level showed better overall survival. [file NIHPPrs4104258V1-supplement-1.pdf]

Supplementary Figure. 1 The overall experimental design

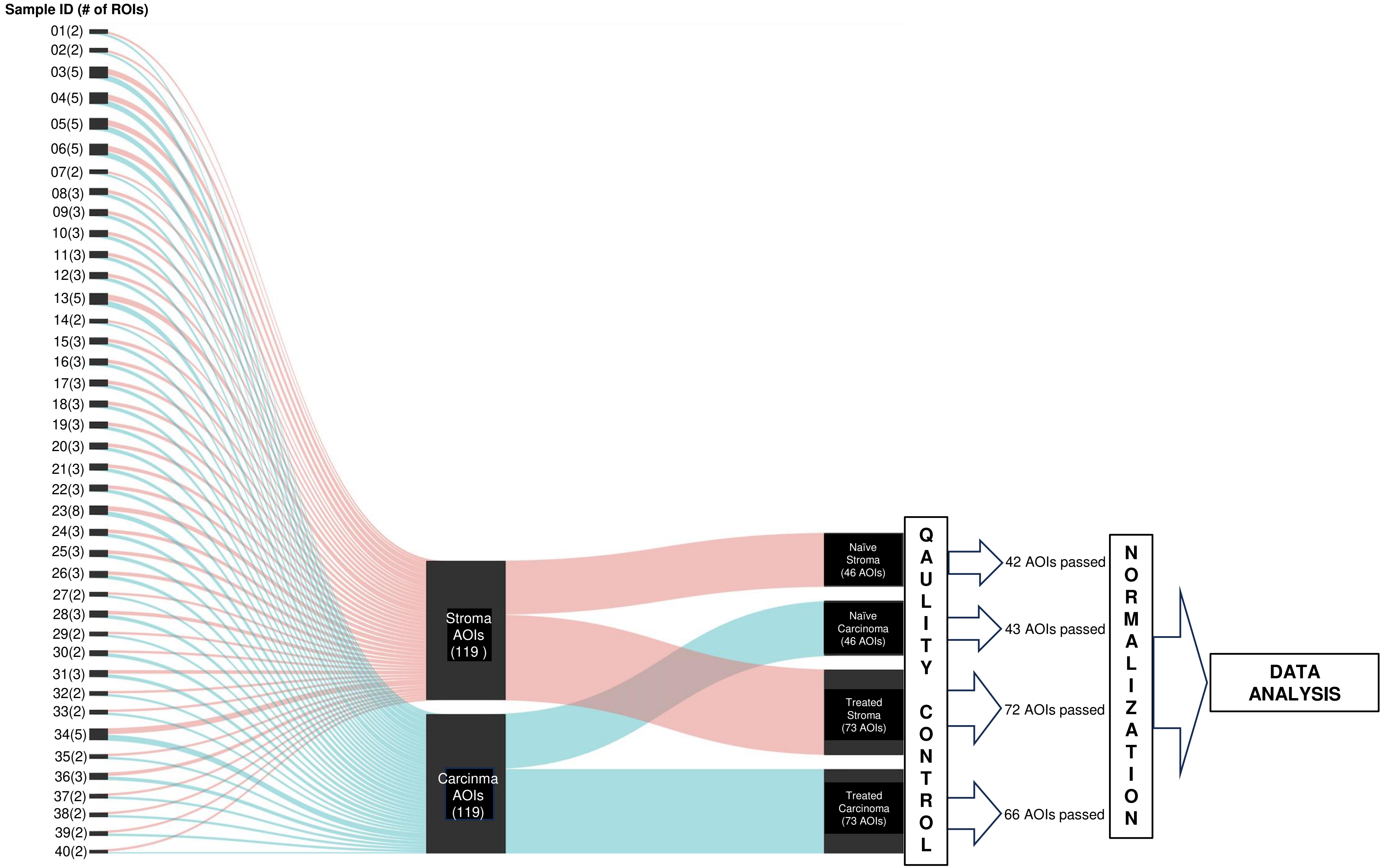

Supplementary Figure. 2 Differentially Expressed Genes (DEGs) in Carcinoma cell AOIs versus the TME AOIs in Naïve group and NAT group

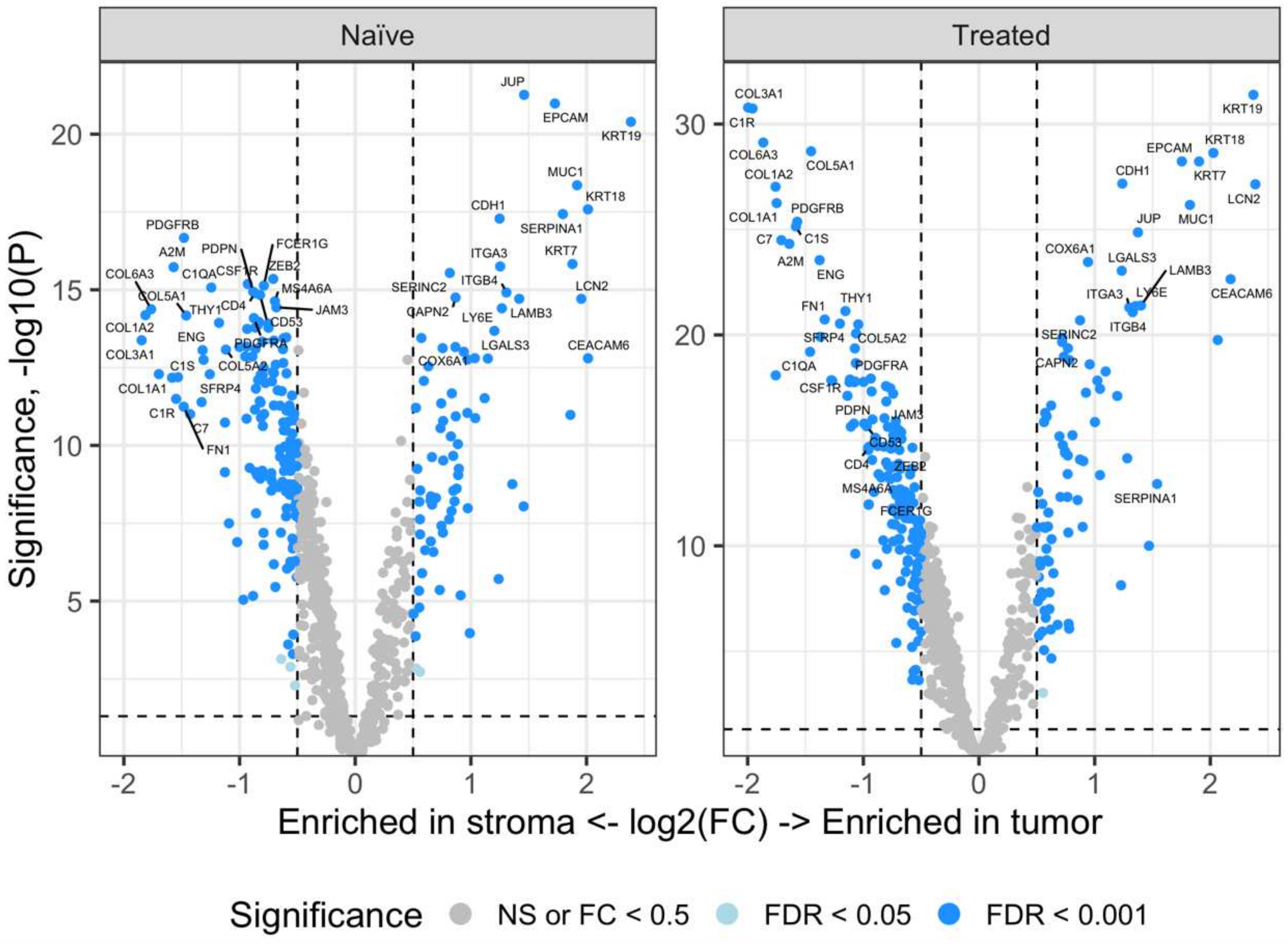

Supplementary Figure. 3 Immune cell distribution in carcinoma and TME AOIs across the study cohort

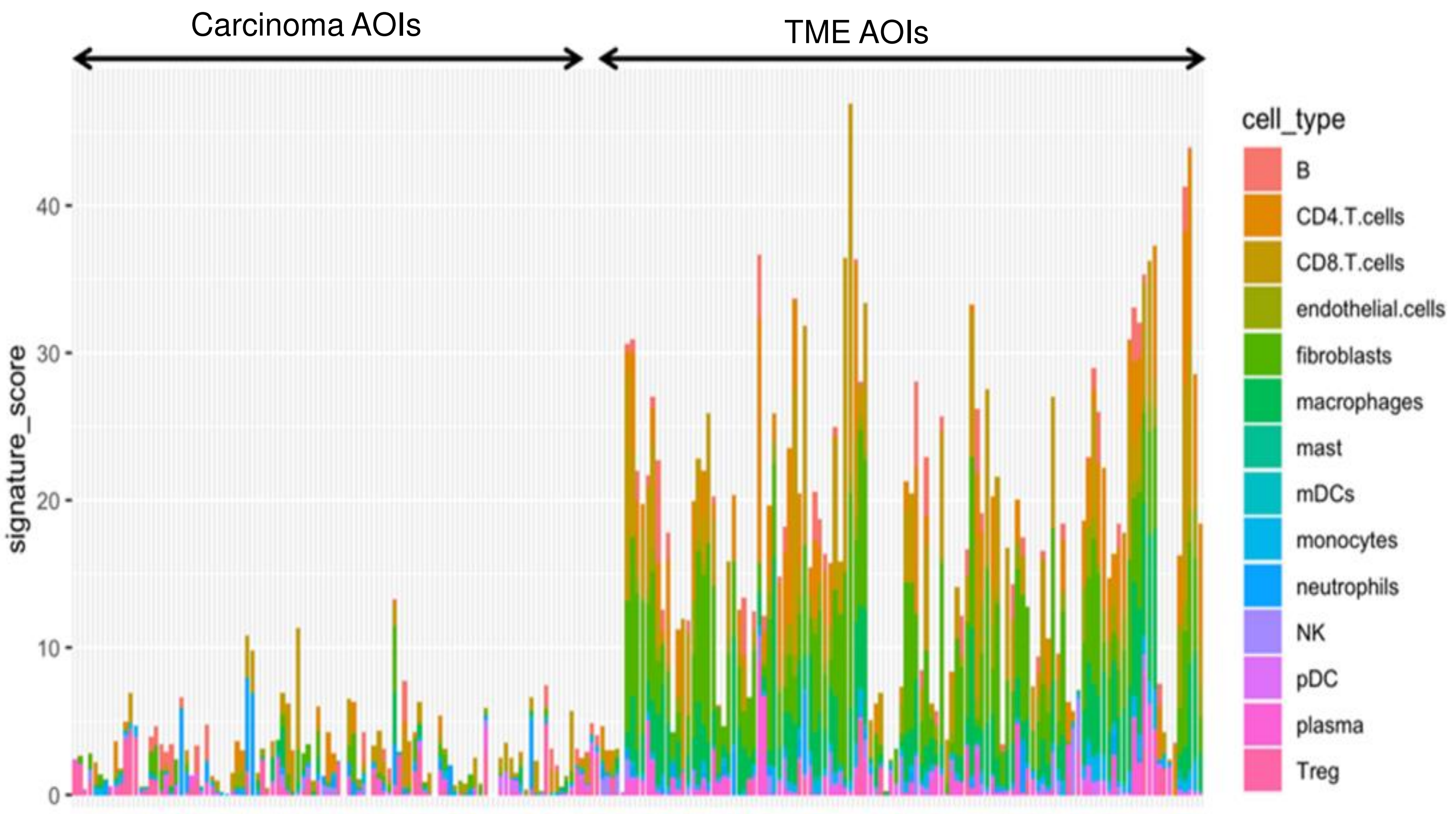

Supplementary Figure. 4 Paired analysis of pre-NAT biopsy and post-NAT resection from four patients

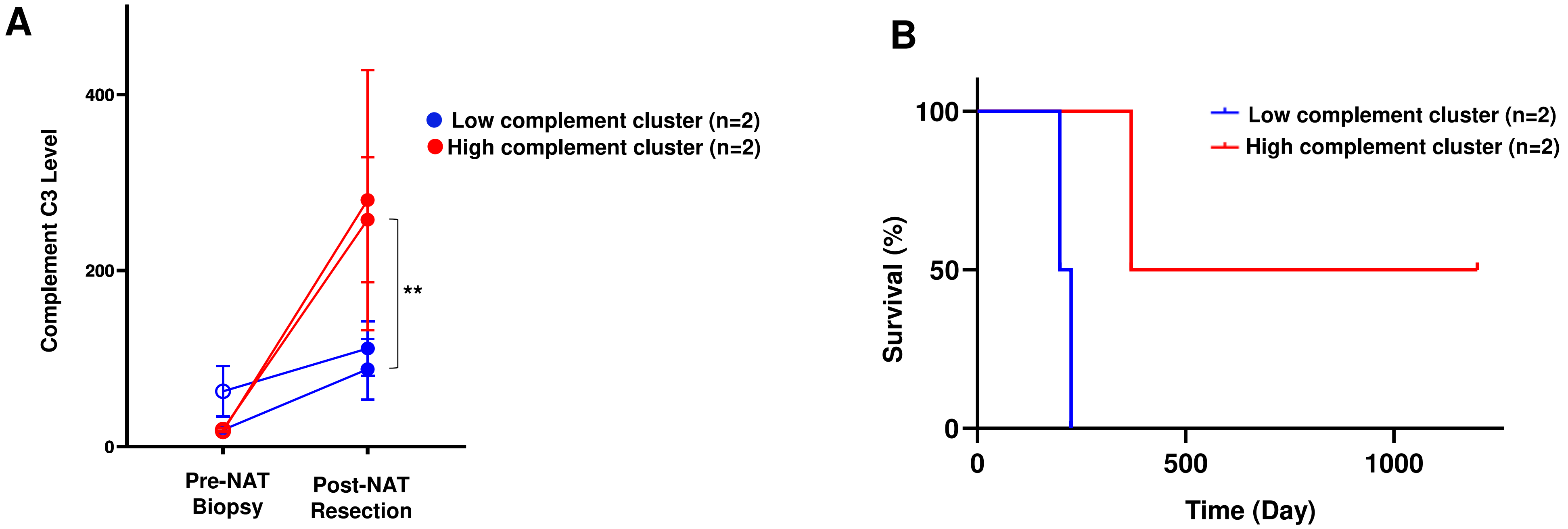

Supplementary Table. 1 Clinicopathologic characteristics of the NAT-naïve patients and NAT-treated patients in our study cohort

Characteristics		All subjects	NAT group	Naïve group
**Total (n)**		36	23	13
**Median age, years (range)**		65.5 (49-86)	66 (59-78)	63 (49-86)
**Age group (years), n (%)**				
	<65	15 (41.7)	8 (34.8)	7 (53.8)
	>=65	21 (58.3)	15 (65.2)	6 (46.2)
**Gender, n (%)**				
	Male	17 (47.2)	9 (39.1)	8 (61.5)
	Female	19 (52.8)	14 (60.9)	5 (38.5)
**Tumor size (range)**				
	0.5 – 2.0	9 (25.0)	7 (30.4)	2 (15.4)
	2.1 – 4.0	20 (55.6)	12 (52.2)	8 (61.5)
	>4.0	7 (19.4)	4 (17.4)	3 (23.1)
**Cancer grading, n (%)**				
	G1	6 (16.7)	3 (13.0)	3 (23.1)
	G2	20 (55.6)	12 (52.2)	8 (61.5)
	G3	10 (27.7)	8 (34.8)	2 (15.4)
**Staging, n (%)**				
	I and II	23 (63.9)	17 (73.9)	6 (46.2)
	III and IV	13 (36.1)	6 (26.1)	7 (53.8)

Supplementary Table. 2
Clinicopathologic characteristics of 4 patients for the comparison between pre-NAT biopsy and post-NAT resection

**Characteristics**		**All subjects**	**High TME C3 group**	**Low TME C3 group**
**Total, n**		4	2	2
**Median age, years (range)**		69.5 (67-73)	68 (67-69)	71.5 (70-73)
**Age group (years), n (%)**				
	<70	2 (50.0)	2 (100.0)	0 (0)
	>=70	2 (50.0)	0 (0)	2 (100.0)
**Gender, n (%)**				
	Male	1 (25.0)	0 (0)	1 (50.0)
	Female	3 (75.0)	2 (100.0)	1 (50.0)
**Tumor size, cm (%)**				
	0.5 – 2.0	1 (25.0)	0 (0)	1 (50.0)
	2.1 – 4.0	2 (50.0)	2 (100.0)	0 (0)
	>4.0	1 (25.0)	0 (0)	1 (50.0)
**Cancer grading, n (%)**				
	G1	0 (0)	0 (0)	0 (0)
	G2	3 (75.0)	2 (100.0)	1 (50.0)
	G3	1 (25.0)	0 (0)	1 (50.0)
**Staging, n (%)**				
	I	1 (25)	1 (25)	1 (25)
	II	3 (75)	3 (75)	3 (75)
